# Metallurgy and Mechanism of Underwater Wet Cutting Using Oxidizing and Exothermic Flux-Cored Wires

**DOI:** 10.3390/ma14164655

**Published:** 2021-08-18

**Authors:** Sergey G. Parshin, Alexey M. Levchenko, Pengfei Wang

**Affiliations:** 1 Institute of Mechanical Engineering, Materials, and Transport, Peter the Great St. Petersburg Polytechnic University, 195251 St. Petersburg, Russia; van2.p@edu.spbstu.ru; 2 Department of Underwater Welding and Technologies, Educational Scientific and Technical Center “Svarka”, 195251 St. Petersburg, Russia; lab@untc-svarka.ru

**Keywords:** underwater wet cutting, flux-cored wire, metallurgical processes, electric arc

## Abstract

This paper considers the metallurgical processes of dissociation, ionization, oxidation, deoxidation, and dissolution of oxides during underwater wet cutting. A multiphase mechanism of underwater wet cutting consisting of working and idle cycles of the electrical process in a pulsating vapor gas bubble is proposed. A model of arc penetration into metal due to metal oxidation and stabilization of the arc by the inner walls of a narrow kerf is proposed. For underwater cutting of 10 KhSND, 304L steel, CuAl5, and AlMg4.5Mn0.7 alloy, we provide a principle of modeling the phase composition of the gas mixture based on high oxygen concentration, improving ionization, enthalpy, heat capacity, and thermal conductivity of plasma through the use of a mixture of KNO_3_, FeCO_3_ and aluminum. The method of improving the thermophysical properties and ionization of plasma due to the exothermic effect when introducing Fe_3_O_4_, MoO_2_, WO_2_ oxides and Al, Mg, Ti deoxidizers is proposed. Although a negative effect of refractory slag was revealed, it could be removed by using the method of reducing surface tension through the ionic dissolution of refractory oxides in Na_3_AlF_6_ cryolite. In underwater cutting of 10 KhSND and 304L, the steel welding current was 344–402 A with a voltage of 36–39 V; in cutting of CuAl5 and AlMg4.5Mn0.7 alloy, the welding current was 360–406; 240 A, with a voltage of 35–37; 38 V, respectively, with the optimal composition of flux-cored wire: 50–60% FeCO_3_ and KNO_3_, 20–30% aluminum, 20% Na_3_AlF_6_. Application of flux-cored wires of the KNO_3_-FeCO_3_-Na_3_AlF_6_-Al system allowed stable cutting of 10KhSND, AISI 304L steels, and CuAl5 bronze with kerf width up to 2.5–4.7 mm.

## 1. Introduction

Underwater welding and cutting are used in the construction and repair of important objects, including offshore oil and gas platforms, wind farms, ports, hydraulic structures, underwater oil and gas pipelines, and the lifting and repair of ships [1]. Underwater welding and cutting are often performed manually by diver–welders using coated and tubular electrodes. Cutting is used to remove defective metal; after cutting, welding is performed on the cut edges of the parts. During underwater wet cutting, steel is thermally affected and saturated with hydrogen. The presence of impurities, slag, and metal hydrogenation worsens the quality of welding and reduces strength, ductility, and impact toughness [2]. Underwater wet welding is complicated by hydrogen-induced cold cracks, porosity, slag inclusions, and delayed hydrogen embrittlement [3,4,5].

The most commonly used methods for underwater cutting are manual arc cutting with coated electrodes, manual oxy-arc cutting with tubular and exothermic electrodes, semi-automatic arc cutting with flux-cored wire, gas-oxygen, automatic plasma cutting, laser cutting, electromechanical cutting, electrohydraulic cutting, explosion cutting, wire cutting, and other methods [6,7,8,9,10], as shown in Table 1.

The mechanism of underwater wet cutting was previously studied in other works [11,12]. Technological properties of flux-cored wires of 2 and 2.4 mm in diameter based on Ba(OH)_2_·8H_2_O and Na_2_SiO_3_ were studied at the current of 450–600 A, voltage 50–55 V, and wire feed rate of 3.5–6 m/min when cutting steel of 10–20 mm in thickness. The authors proposed a scheme of underwater wet cutting, according to which the flux-cored wire during cutting deepens into the metal and forms a kerf.

The analysis of patents for inventions shows a great variety of chemical compositions of flux-cored wires. One of the wires with the filling factor of 16–18% has the composition, wt.%: 40–60 saltpeter (sodium nitrate), 30–40 barium hydroxide, iron powder—the rest [13]. In another patent, a flux-core wire with the filling factor of 16–20% was developed, with the composition, wt.%: 70–80 iron carbonate, 17–23 barium hydroxide, and 3–7 alkaline silicate [14]. There is a wire with the composition, wt.%: 20–30 barium peroxide; 20–50 iron oxide; 10–20 K_2_Cr_2_O_7_; 5–15 aluminum; 5–40 Teflon [15]. Another wire with the filling factor of 25–30% had the composition, wt.%: 70–75 iron oxide; 21–24 aluminum; 4–6 graphite [15].

A Chinese patent provides a wire with the composition, wt.%: 40–60 aluminum and magnesium thermite, 10–35 marble, 5–10 potassium carbonate, 1–2 rare-earth element, 0–20 iron powder, 0–10 nickel powder, 0–15 potassium chlorate, and 5–10 hydroxymethylcellulose [16]. A 2 mm diameter wire was tested while cutting 8 mm thick Q235 steel at the current of 150–290 A, voltage 30–40 V. There is a flux-cored wire with the composition, wt.%: 8–24 dolomite, 0.5–4 quartz sand, 0.1–3 cesium carbonate, 0.1–3 potassium carbonate, 0.6–2.4 cerium oxide, 2.4–4.8 manganese peroxide, and calcium peroxide or barium peroxide—the rest [17]. Tests of a 2.2 mm diameter wire were conducted while cutting 20 mm thick Q235 steel at the current of 450–500 A, voltage of 45–50 V, and cutting speed of 200–230 mm/min.

In underwater wet cutting, the high stability of arc burning is important. For current and voltage studies, a 2 mm diameter oxidizing wire based on carbonates (MgCO_3_, BaCO_3_) and peroxides (BaO_2_, CaO_2_) and an exothermic wire with 75% termite mixture (Al + CuO) with the filling factor of 25–26% were used [18]. An 8 mm thick Q235 steel was used for cutting at the wire feed rate of 8–17 m/min, current of 390–510 A, arc voltage of 35–40 V, and cutting speed of 270–300 mm/min. Deviation of the current reached ±100–238 A, and for voltage, ±1.4–3.8 V. Kerf width on the upper surface was 3.8–8.8 mm with a deviation of ±1.2–1.9 mm; on the lower surface, 6.1–12.1 mm. According to the authors, the underwater wet cutting mechanism has four modes: normal arc, long arc, short arc, and exit of the wire from the kerf.

Underwater wet cutting of 6 mm thick steel with a 2 mm PPR-AN2 flux-cored wire was studied using a high-speed video camera at 2000 fps at a current of 550 A and voltage of 25 V [19]. During cutting, there was periodic ignition and deepening the arc into the metal with slag formation. According to the authors, cutting has the modes: short circuit, arc development, and expansion of the vapor gas bubble, deepening the arc into the metal.

Cutting mechanism with a 2 mm diameter PPR-AN2 flux-cored wire was studied when cutting 16 mm thick Q235 steel in air and underwater [7,20]. The authors found four modes: arc burning inside the kerf, exit of the wire from the kef below the metal surface, short-circuiting of the wire against the edge of the metal, and re-ignition of the arc.

Temperature fields and the mechanism of underwater cutting of 16 mm thick Q235 steel were studied using an oxidizing flux-cored wire based on dolomite, cellulose, and peroxide [21]. The wire diameter was 2.2 mm, the current strength was 350 A, the voltage was 40 V, the wire feed rate was 4.5 m/min, the cutting speed was 130 mm/min, and the kerf width was 8–11 mm. Studies revealed the periodicity of arc ignition and breaking. However, no data on short circuits, duration of working, and idle cycles are available. According to the authors, cutting occurs at a constant arc length. However, this conclusion contradicts the oscillogram, according to which the current decreases and the voltage increases during cutting. These data indicate that the arc lengthens during cutting.

Parshin et al. [22] investigated metallurgical processes, but only in underwater welding. Parshin et al. [23] investigated electrical parameters in underwater cutting but without metallurgical processes. Parshin and Levchenko [24] also provided short information on underwater welding and cutting using flux-cored wire with the complex KOPS-M without a detailed study of metallurgical processes.

Therefore, the analysis of research on underwater wet cutting with a flux-cored wire shows that there is fragmentary information about the mechanism of cutting, arc properties, and slag formation. Detailed studies of metallurgical processes in the gas phase, ionized plasma, and slag are needed. This makes the research relevant.

The aim of the work was to study the mechanism and metallurgical processes of underwater wet cutting using flux-cored wires.

## 2. Materials and Methods

**Figure 1:**
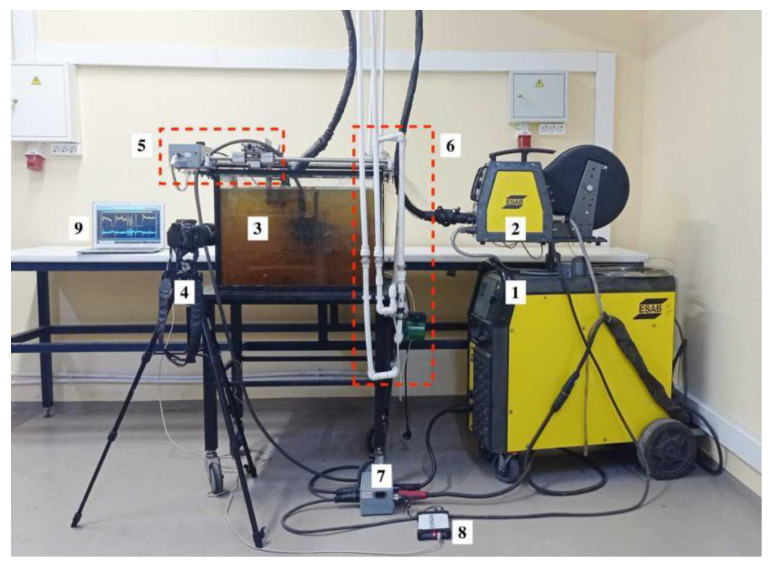
Underwater wet cutting setup: (**1**) source of power; (**2**) wire feed system; (**3**) water tank; (**4**) video camera; (**5**) automat of torch movement; (**6**) water supply system and pump; (**7**) welding current and arc voltage sensors; (**8**) USB-oscilloscope; (**9**) laptop.

For underwater wet cutting, plates in size of 200 mm × 100 mm × 10 mm of 10KhSND low alloy steel according to GOST 19281-2014 (in Russian) or S355J0WP according to EN 10025-2:2004, wt.%: 0.08C; 1Si; 0.6Mn; 0.8Ni; 0.8Cr; 0.1V; 0.5Cu, AISI 304L of 16 mm according to ASTM A240/A240M-20a, wt.%: 0.01C; 0.6Si; 1.8Mn; 10 Ni; 18Cr, CuAl5 aluminum bronze of 10 mm according to ISO 428:1983, wt.%: 5Al; 0.3Fe; 0.7Ni; 0.4Mn; 0.3Zn and AlMg4.5Mn0.7 alloy of 6 and 12 mm according to EN AW-5083 were used. Underwater wet automatic cutting was carried out in a laboratory setup using flux-cored wires PPR-APL1 (UNTC “Svarka”, St. Petersburg, Russia) with a diameter of 2 mm in accordance with ISO 12224-1 and GOST 26271-84 (in Russian), with the filling factor of 22%, as shown in Figure 1.

For tests, we used 3 compositions of experimental flux-cored wires with different contents of the oxidizing mixture, as shown in Table 2.

The power source was ESAB Origo MIG L405 with idling voltage 45 V. The immersion depth of the samples in freshwater was 300 mm. Underwater cutting parameters are shown in Table 3.

Arc voltage and current were measured using a digital USB oscilloscope and Multi VirAnalyzer 3.10.3.6 software (Harbin Instrustar Electronic Technology Co., Ltd., Harbin, China) at a frequency of 32 kHz. Analysis of electrical parameters from oscillograms was performed using the MATLAB program (The MathWorks, Inc., Natick, MA, USA). Research of the vapor gas bubble formation was performed by the shadow method with a laser system and Phantom VEO 710L high-speed camera (Vision Research, Wayne, NJ, USA) with a frequency of 8000 Hz. Phantom CV 2.7.756.2 software (Vision Research Inc., Wayne, NJ, USA) was used for image visualization and analysis. For thermodynamic modeling of the chemical reactions, the plasma thermophysical properties and the phase composition of plasma and slag used the IVTANTHERMO 2001 software (Joint Institute for High Temperatures of the Russian Academy of Sciences, Moscow, Russia) and FactSage 8.1 software (CRCT, Montreal, QC, Canada).

## 3. Research Results

### 3.1. Mechanism of Underwater Wet Cutting

**Figure 2:**
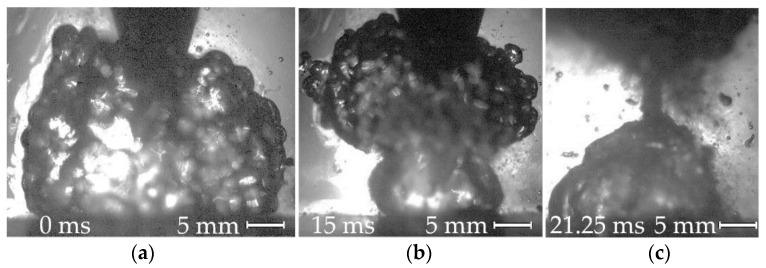
Dynamics of the vapor gas bubble during underwater wet cutting using flux-cored wire: (**a**) arc deepening in the metal, vapor gas bubble stabilization; (**b**) vapor gas bubble collapse and new bubble formation; (**c**) old bubble detachment, new bubble expansion.

Underwater cutting, as with welding, takes place in a vapor gas bubble atmosphere, as shown in Figure 2.

**Figure 3:**
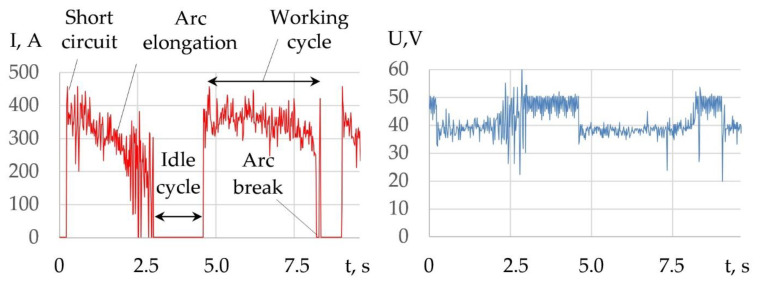
Typical oscillogram of current and voltage during underwater cutting with a 2 mm diameter PPR-APL1 flux-cored wire.

The peculiarity of underwater wet cutting is that when the components decompose, oxygen is produced, which oxidizes the iron and forms slag. The slag is then forced out of the cutting zone under arc pressure. Another feature is the presence of periodic working and idle cycles in the electrical process. These cycles are associated with periodic ignition and breaking of the electric arc during the formation of a through kerf, as shown in Figure 3.

**Figure 4:**
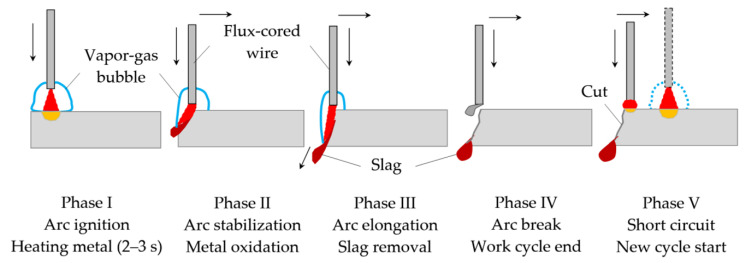
Mechanism of underwater wet cutting using flux-cored wires.

The working cycle of cutting includes the physical phenomena: short-circuit, arc elongation, and arc break. The idle cycle is the pause between arc break and short-circuit. Depending on the cutting mode and composition of the flux-cored wire, the duration of working cycles is 1.5–5 s, and idle cycles is 0.6–1.7 s. The mechanism of the underwater wet cutting process consists of five phases, as shown in Figure 4.

The mechanism of underwater wet cutting has five phases: arc starting, metal heating, vapor gas bubble formation; arc stabilization, beginning of metal oxidation, vapor gas bubble expansion; arc elongation, slag removal, vapor gas bubble collapse; arc breaking with the formation of a through kerf, idle cycle; short circuit and the start of a new working cycle. The cutting process begins with phase I starting the arc, forming a vapor gas bubble, and heating the metal to initiate the oxidation reaction. Stabilization of the arc and development of the oxidation reaction in phase II leads to elongation of the arc as the arc deepens into the kerf, increasing arc voltage and slag formation. In phase III, there is a further increase in arc voltage and arc length, a decrease in the current, formation of a through kerf, and removal of slag by arc pressure. At the point of formation of the through kerf, the arc breaks, and the working cycle ends in phase III. The pause between phases IV and V is an idle cycle in which there is zero current and voltage equals the output voltage of the power supply. A short circuit at the cutting edge begins a new working cycle in phase V.

**Figure 5:**
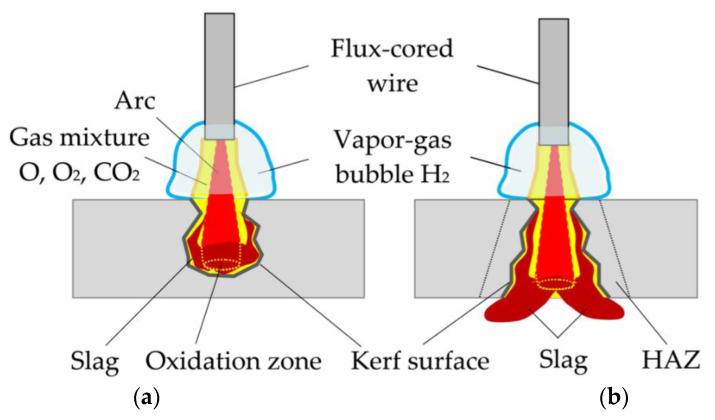
Model of arc penetration and stabilization during underwater wet arc cutting using a flux-cored wire in the cross-section: (**a**) closed kerf; (**b**) open kerf. (HAZ) Heat-affected zone.

Underwater wet cutting is similar to welding because it takes place in a vapor gas bubble with an intense decomposition of water and flux-cored wire components. However, unlike welding, the cutting process takes place in a narrow kerf that stabilizes arc burning and the process of plasma ionization in the oxidizing gas mixture, as shown in Figure 5.

### 3.2. Dissociation of Components and Oxidation of Iron

**Figure 6:**
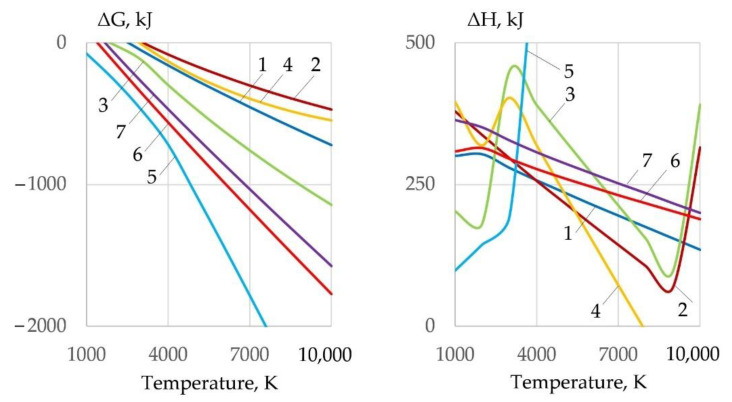
Change of Gibbs free energy and enthalpy of salt dissociation reactions (**1**)–(**7**).

The formation of an oxidizing atmosphere around the arc is a prerequisite for underwater cutting. The composition of the oxidizing atmosphere is determined by the dissociation of the components of the flux-cored wire. Alkali metal and iron carbonates, as well as sodium and potassium nitrates, are used as basic components. In endothermic Reactions (1)–(7), dissociation of salts and formation of CO_2_, O_2_, and NO occurs, as shown in Figure 6:

Na_2_CO_3_ = Na_2_O + CO_2_ − Q (1)

K_2_CO_3_ = K_2_O + CO_2_ − Q (2)

Li_2_CO_3_ = Li_2_O + CO_2_ − Q (3)

Cs_2_CO_3_ = Cs_2_O + CO_2_ − Q (4)

FeCO_3_ = FeO + CO_2_ − Q (5)

NaNO_3_ = 0.5Na_2_O + NO +0.5O_2_ − Q (6)

KNO_3_ = 0.5K_2_O + NO +0.5O_2_ − Q (7)

Dissociation reactions require energy, so the change in enthalpy of Reactions (1)–(7) has a positive value. Particularly, active dissociation occurs in FeCO_3_, NaNO_3_, KNO_3_, and Li_2_CO_3_ salts, of which FeCO_3_ and Li_2_CO_3_ have minimum ΔH values at 1000–3000 K. Formation of oxidizing atmosphere during cutting leads to oxidation of Fe, Cr, Ni by Reactions (8)–(14):

Fe + O = FeO + Q(8)

Fe + 0.5O_2_ = FeO + Q (9)

Fe + CO = FeO + C ± Q(10)

Fe + CO_2_ = FeO + CO − Q (11)

Fe + NO = FeO + 0.5N_2_ + Q (12)

Cr + 1.5O = 0.5Cr_2_O_3_ + Q (13)

Ni + O = NiO + Q(14)

At the temperature of 1000–4000 K, iron oxidation reactions are possible (15)–(21) when interacting with salts, especially with FeCO_3_, NaNO_3_, KNO_3_:

Na_2_CO_3_ + Fe = FeO + Na_2_O + CO (15)

K_2_CO_3_ + Fe = FeO + K_2_O + CO (16)

Li_2_CO_3_ + Fe = FeO + Li_2_O + CO (17)

Cs_2_CO_3_ + Fe = FeO + Cs_2_O + CO (18)

FeCO_3_ + Fe = FeO + CO_2_ (19)

NaNO_3_ + Fe = 0.5Fe_2_O_3_ + 0.5Na_2_O + NO (20)

KNO_3_ + Fe = 0.5Fe_2_O_3_ + 0.5K_2_O + NO (21)

**Figure 7:**
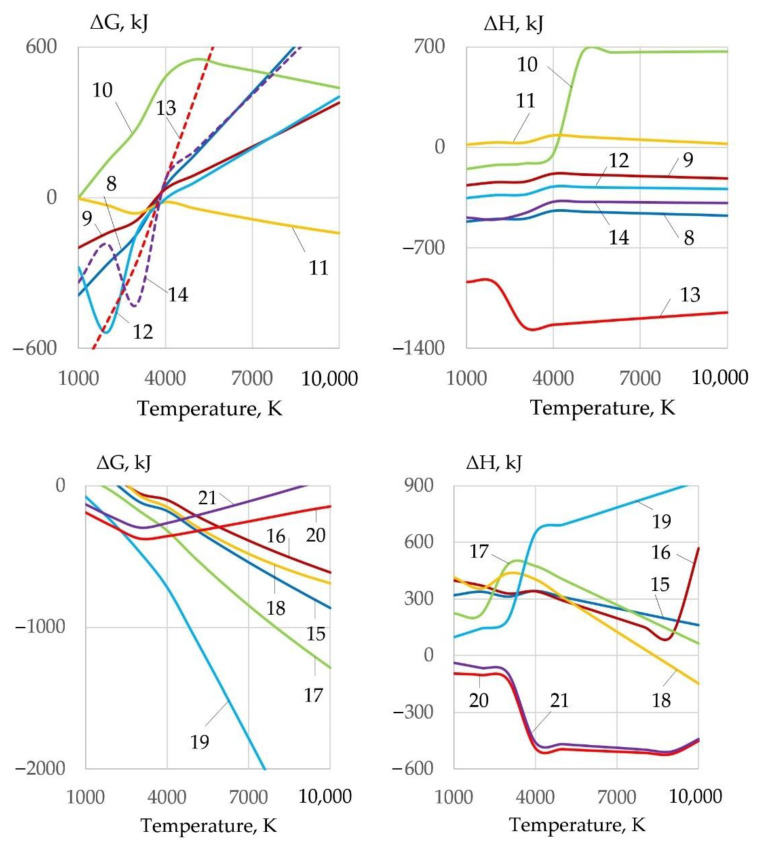
Change of Gibbs free energy and enthalpy of iron oxidation reactions (**8**)–(**21**) in interaction with gases and salts.

In the oxidation reactions of Fe, Cr, and Ni, the most active components are O, O_2_, CO_2_, and NO. Unlike dissociation reactions, oxidation reactions release heat, i.e., reduce the internal energy of the gas system, as shown in Figure 7.

**Figure 8:**
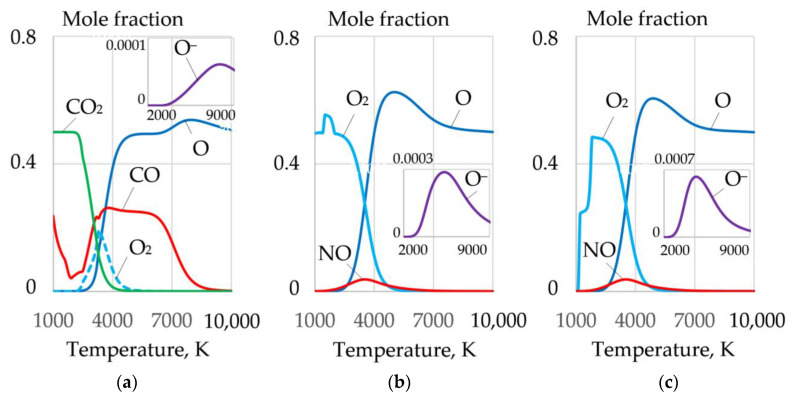
Mole fractions of gases and negative ions of oxygen O^−^ during dissociation of salts: (**a**) FeCO_3_; (**b**) NaNO_3_; (**c**) KNO_3_.

Dissociation of salts leads to the formation of a complex mixture of gases O, O_2_, CO, CO_2_, and NO, as shown in Figure 8.

**Figure 9:**
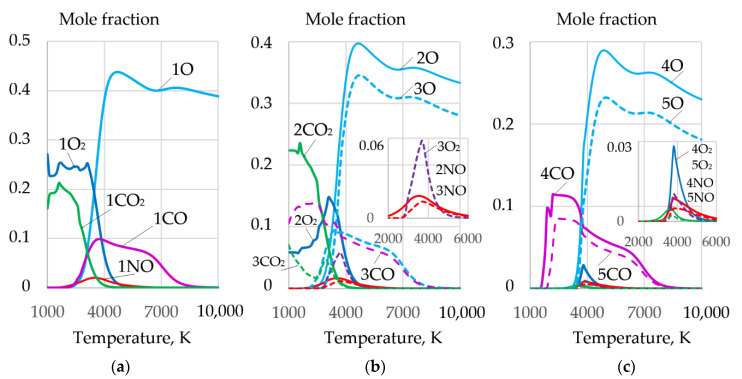
Mole fractions of gases in the system, wt.%: (**a**): (**1**) 40FeCO_3_ + 40KNO_3_ + 20Na_3_AlF_6_; (**b**): (**2**) 35FeCO_3_ + 35KNO_3_ + 20Na_3_AlF_6_ + 10Al; (**3**) 30FeCO_3_ + 30KNO_3_ + 20Na_3_AlF_6_ + 20Al; (**c**): (**4**) 25FeCO_3_ + 25KNO_3_ + 20Na_3_AlF_6_ + 30Al; (**5**) 20FeCO_3_ + 20KNO_3_ + 20Na_3_AlF_6_ + 40Al.

**Figure 10:**
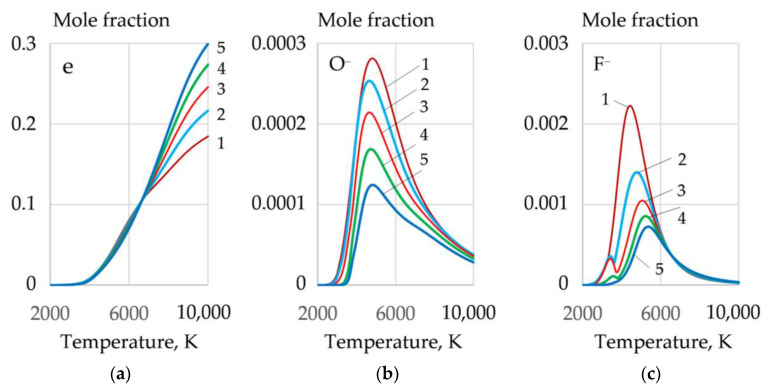
Mole fractions of electrons (**a**), negative ions of atomic oxygen (**b**), and negative ions of fluorine (**c**) in the equilibrium gas system, wt.%: (**1**) 40FeCO_3_ + 40KNO_3_ + 20Na_3_AlF_6_; (**2**) 35FeCO_3_ + 35KNO_3_ + 20Na_3_AlF_6_ + 10Al; (**3**) 30FeCO_3_ + 30KNO_3_ + 20Na_3_AlF_6_ + 20Al; (**4**) 25FeCO_3_ + 25KNO_3_ + 20Na_3_AlF_6_ + 30Al; (**5**) 20FeCO_3_ + 20KNO_3_ + 20Na_3_AlF_6_ + 40Al.

The greatest amount of molecular and atomic oxygen is produced during the dissociation of sodium and potassium nitrate. However, a high concentration of these salts reduces the arc stability because of the formation of negative ions of atomic and molecular oxygen^−^, at the arc boundary. A mixture of FeCO_3_ and KNO_3_ can be used to improve arc stability. Such a mixture provides high oxygen content and low concentration of atomic oxygen (O^−^), as shown in Figure 9 and Figure 10.

### 3.3. Ionization and Thermophysical Properties of Plasma

During cutting, periodic short circuits, breaks, and ignitions of the arc occur. Therefore, the arc plasma should have a high concentration of electrons. Aluminum with low ionization energy—577.6 kJ/mol—can be introduced to improve ionization. As the aluminum content increases, the concentration of electrons increases, and the concentration of F^−^, O^−^ ions decreases, as shown in Figure 10.

**Figure 11:**
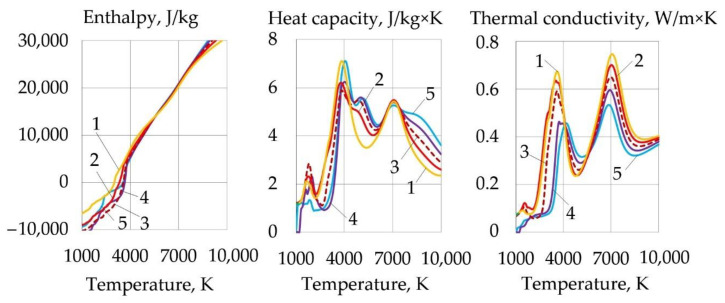
Thermophysical properties of plasma for component mixtures, wt.%: (**1**) 40FeCO_3_ + 40KNO_3_ + 20Na_3_AlF_6_; (**2**) 35FeCO_3_ + 35KNO_3_ + 20Na_3_AlF_6_ + 10Al; (**3**) 30FeCO_3_ + 30KNO_3_ + 20Na_3_AlF_6_ + 20Al; (**4**) 25FeCO_3_ + 25KNO_3_ + 20Na_3_AlF_6_ + 30Al; (**5**) 20FeCO_3_ + 20KNO_3_ + 20Na_3_AlF_6_ + 40Al.

Dissociation of the components causes an increase in the thermal conductivity, heat capacity, and enthalpy of the gas system, and a decrease in dynamic viscosity in the dissociation temperature range of 1000–4000 K, as shown in Figure 11.

As the temperature and salt concentration increase, the enthalpy, heat capacity, and thermal conductivity of the plasma increase. The thermophysical properties of the plasma in the temperature range of 1000–4000 K, in which oxidation reactions have a negative Gibbs free energy, are important for efficient cutting. Increasing the aluminum concentration leads to a slight decrease in enthalpy, heat capacity, and thermal conductivity. The introduction of FeCO_3_ together with KNO_3_ allowed increasing the thermal conductivity of the plasma from 0.59 to 0.67 W/m·K at 3600 K compared to pure KNO_3_.

### 3.4. Exothermic Effect

The exothermic effect with the addition of aluminum improves the stability and increases the weld penetration in underwater wet welding [25]. Underwater wet cutting has significant energy consumption for water and component dissociation, plasma ionization, maintaining stable vapor gas bubble dynamics, and compensating for heat loss as the metal cools in the water.

The arc is a complex self-regulating system according to the principles of Le Chatelier and Steinbeck. To compensate energy loss, the arc increases voltage and power, which reaches 10–20 kW during cutting. Increasing the voltage is also necessary to improve plasma ionization as the arc lengthens in the kerf. The maximum arc length when cutting with a 2 mm flux-cored wire reaches up to 20–25 mm, which is 10 times the length of the welding arc in the air.

**Figure 12:**
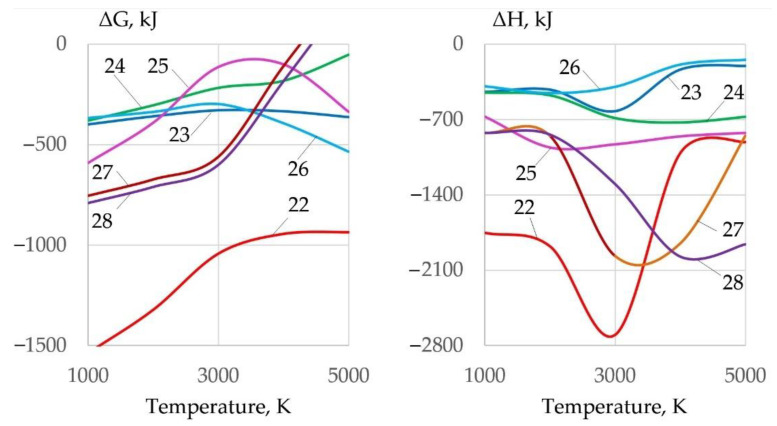
Change of Gibbs free energy and the enthalpy of deoxidation reactions during interaction of oxides with aluminum.

Energy loss and arc elongation require improvement of ionization and thermophysical properties of plasma. Therefore, the gas system must have maximum enthalpy, heat capacity, and thermal conductivity. Heat release in exothermic reactions changes the thermophysical properties of the gas phase. The most efficient oxides for exothermic Reactions (22)–(28) with aluminum are Fe_3_O_4_, MoO_2_, and WO_2_, as shown in Figure 12:

1.5Fe_3_O_4_ + 4Al = 2Al_2_O_3_ + 4.5Fe + Q (22)

0.5Fe_2_O_3_ + Al = 0.5Al_2_O_3_ + Fe + Q (23)

1.5FeO + Al = 0.5Al_2_O_3_ + 1.5Fe + Q (24)

0.5Fe_3_O_4_ + 2Mg = 2MgO + 1.5Fe + Q (25)

0.5Fe_3_O_4_ + Ti = TiO_2_ + 1.5Fe + Q (26)

1.5MoO_2_ + 2Al = Al_2_O_3_ + 1.5Mo + Q (27)

1.5WO_2_ + 2Al = Al_2_O_3_ + 1.5W + Q (28)

**Figure 13:**
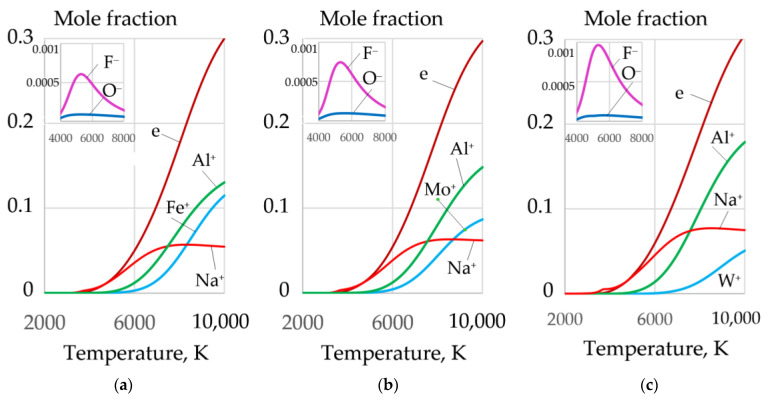
Mole fractions of electrons and ions in the system, wt.%: (**a**) 60Fe_3_O_4_ + 20Al + 20Na_3_AlF_6_; (**b**) 60MoO_2_ + 20Al + 20Na_3_AlF_6_; (**c**) 60WO_2_ + 20Al + 20Na_3_AlF_6_.

The use of an exothermic mixture compared to an oxidizing mixture improves plasma ionization and reduces the concentration of negative F^−^, O^−^ ions, as shown in Figure 13.

**Figure 14:**
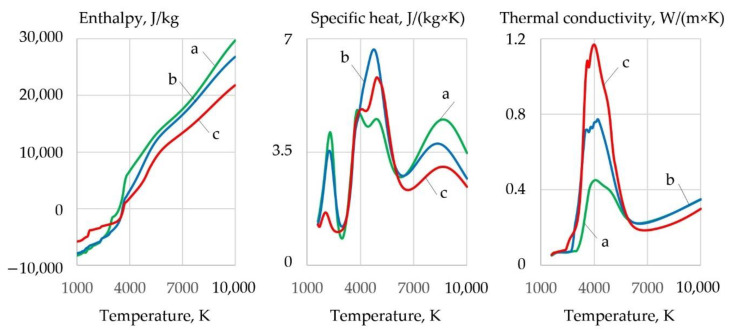
Thermophysical properties of the system, wt.%: (**a**) 60Fe_3_O_4_ + 20Al + 20Na_3_AlF_6_; (**b**) 60MoO_2_ + 20Al + 20Na_3_AlF_6_; (**c**) 60WO_2_ + 20Al + 20Na_3_AlF_6_.

However, the enthalpy, heat capacity, and thermal conductivity are reduced compared to the oxidizing mixture, wt.%: 30FeCO_3_ + 30KNO_3_ + 20Na_3_AlF_6_ + 20Al, as shown in Figure 11 and Figure 14.

### 3.5. Formation of Oxides and Slag

**Figure 15:**
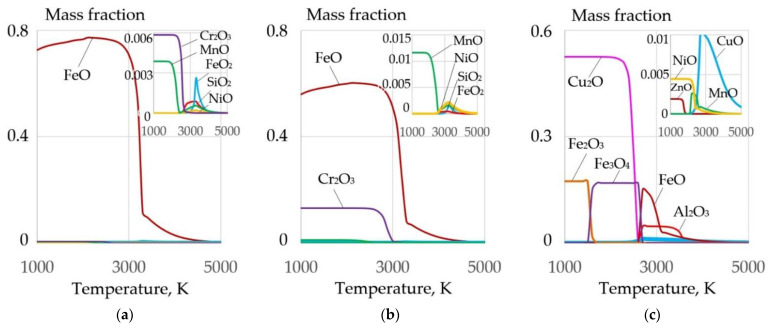
Mass fractions of oxides in the equilibrium system, wt.%: (**a**) 25FeCO_3_-25KNO_3_-50(10KhSND) steel; (**b**) 25FeCO_3_-25KNO_3_-50(AISI 304L) steel; (**c**) 25FeCO_3_-25KNO_3_-50(CuAl_5_) alloy.

Underwater wet cutting can be used for various steels and alloys that contain the elements Fe, Si, Mn, Cr, Ni, Cu, Al, and Ti. When the metal is oxidized, a refractory slag is formed that hinders the cutting process, as shown in Figure 15.

**Figure 16:**
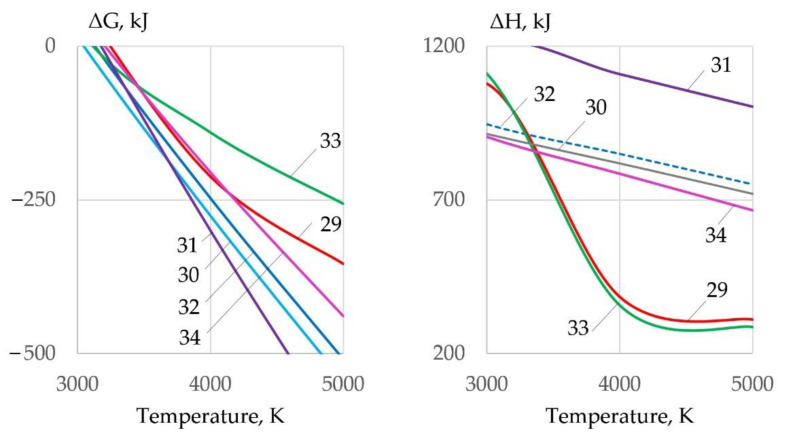
Change of Gibbs free energy and enthalpy of reactions (**29**–**34**) of the interaction of oxides with cryolite.

The slag is removed from the cutting zone under the influence of the balance of forces: gravitational force, arc pressure force, and surface tension force. To remove the slag, it is necessary to reduce its surface tension, which can be achieved by ionic dissolution of oxides in Na_3_AlF_6_ cryolite. At high temperatures (in the arc zone), interaction of slag oxides with cryolite is possible by Reactions (29)–(34), as shown in Figure 16:

1.5FeO + Na_3_AlF_6_ = 1.5FeF_2_ + 1.5Na_2_O + AlF_3_ (29)

0.5Fe_2_O_3_ + Na_3_AlF_6_ = FeF_3_ + 1.5Na_2_O + AlF_3_ (30)

0.5Fe_3_O_4_ + Na_3_AlF_6_ = 1.5FeF_2_ + 1.5Na_2_O + AlF_3_ + 0.5O_2_ (31)

0.5Cr_2_O_3_ + Na_3_AlF_6_ = CrF_3_ + 1.5Na_2_O + AlF_3_ (32)

1.5NiO + Na_3_AlF_6_ = 1.5NiF_2_ + 1.5Na_2_O + AlF_3_ (33)

0.5Al_2_O_3_ + Na_3_AlF_6_ = 2AlF_3_ + 1.5Na_2_O (34)

Endothermic reactions of oxides dissolution in cryolite are possible because the reactions have a negative Gibbs free energy. Spectral analysis of the Na_3_AlF_6_-FeO-Fe_2_O_3_ slag system confirm the interaction of iron oxides with cryolite with the formation of iron fluorides and oxyfluoroaluminates, which reduce the surface tension of molten slag [26]. Similar data are presented in [27], which confirms the formation of FeF_2_, FeF_3_, and Al_2_O_3_ fluorides during the interaction of iron oxides with cryolite. Thermodynamic modeling of dissolution of refractory chromium oxide Cr_2_O_3_ in Na_3_AlF_6_ is considered in [28,29]. Dissolution of Cr_2_O_3_ occurs through the interaction with cryolite with the formation of fluoride compounds of chromium and aluminum. Dissolution of NiO in cryolite also occurs through reactions with the formation of nickel fluorides and aluminum oxide [30]. Dissolution of oxides CuO, Cu_2_O, and TiO_2_ occurs through the interaction with liquid cryolite with the formation of fluorides CuF and CuF_2_ and oxyfluoride NaTiOF_3_ [31,32].

### 3.6. Testing of Flux-Cored Wires

**Figure 17:**
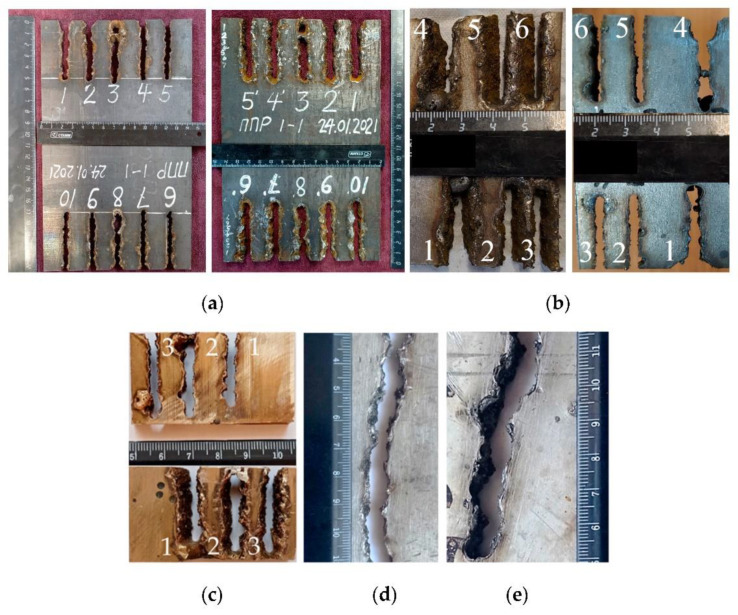
Outside and backside of the samples after cutting: (**a**) 10KhSND steel of 10 mm, automatic cutting with PPR-APL1-1; (**b**) AISI 304L steel of 16 mm, automatic cutting with PPR-APL1-1; (**c**) CuAl5 alloy of 10 mm, automatic cutting with PPR-APL1-2; (**d**,**e**) AlMg4.5Mn0.7 of 6 and 12 mm, semi-automatic cutting with PPR-APL1-3, current of 240 A, voltage of 38 V.

Flux-cored wires based on FeCO_3_-KNO_3_-Na_3_AlF_6_-Al are effective for cutting various steels and alloys up to 16 mm thick and potentially over, as shown in Figure 17.

**Figure 18:**
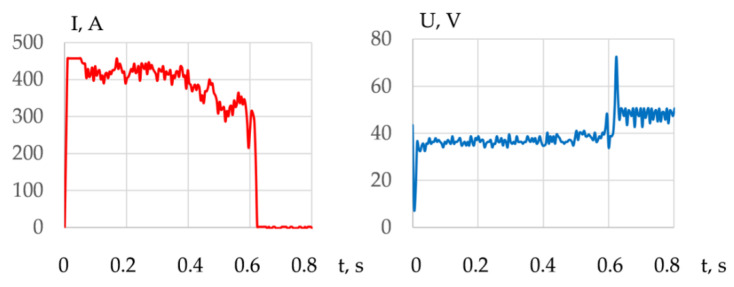
Typical change in the current and voltage during a working cycle when cutting 10 KhSND steel with PPR-APL1-1 wire for cut no. 6.

A decrease in the current in the working cycle and an increase in the voltage confirm an increase in the arc length as the arc deepens into the metal, as shown in Figure 18.

As the wire feed rate increases, the current increases, the short-circuit frequency increases, and the voltage decreases. As the cutting travel speed decreases, the current increases, probably due to the improved arc stability and plasma ionization. The value of current and voltage deviations decreases as the wire feed rate and cutting travel speed increase, as shown in Table 4, Table 5 and Table 6.

**Figure 19:**
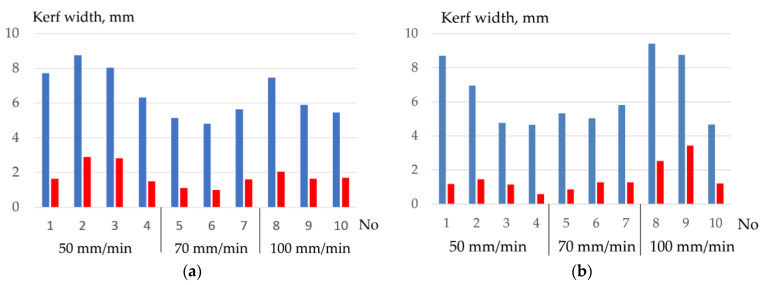
Variation of the average cutting width and deviations when cutting 10 KhSND steel at speeds of 50, 70, and 100 mm/min: (**a**) PPR-APL1-1 flux-cored wire; (**b**) PPR-APL1-2 flux-cored wire.

When cutting 10KhSND steel with flux-cored wires PPR-APL1-1 and PPR-APL1-2 at the cutting speed of 50–70 mm/min at the wire feed rate of 9–10 m/min, the maximum current of 391–420 A with a deviation of ±20–50 A, the minimum voltage of 36.3–37.8 V with deviations ±1.5–3.2 V is reached. The analysis of the kerf width and electrical parameters showed that the best stability of cutting 10KhSND steel is achieved at the concentration of the salt mixture at 50–60% and aluminum at 20–30%, as shown in Figure 19.

The hardness measurement method was used to determine the width of the HAZ in the underwater wet cutting of steels [33,34]. The results of measuring the hardness and HAZ width are shown in Table 7.

The minimal HAZ width is achieved in the underwater cutting of steels with 50% salt mixture and 30% aluminum in flux-cored wire.

## 4. Conclusions

1. The mechanism of underwater wet cutting with a flux-cored wire consists of the formation of a kerf in the oxygen atmosphere around the arc’s active spot. The formation of the kerf occurs within a pulsating vapor gas bubble under the influence of working cycles with the duration of 1.5–5 s and idle cycles with the duration of 0.6–1.7 s. In underwater cutting of 10 KhSND and 304L steel welding, the current was 344–402 A, the voltage was 36–39 V; in cutting of CuAl5 and AlMg4.5Mn0.7 alloy, the welding current was 360–406; 240 A and the voltage was 35–37; 38 V, respectively, with the optimal composition of flux-cored wire: 50–60% FeCO_3_ and KNO_3_, 20–30% aluminum, 20% Na_3_AlF_6_.
2. A model of arc penetration and stabilization during underwater wet cutting using flux-cored wires is proposed. The model implies that the formation of the kerf occurs through penetration of the arc into the metal due to oxidation of the metal in a narrow zone around the arc’s active spot. The walls of the kerf mechanically compress the arc column, which stabilizes the arc and allows the arc to increase its length as it cuts. The compression of the arc by the walls of the kerf causes increasing ionization, temperature, and pressure of the arc.
3. When metals oxidize, refractory molten slag from oxides of FeO, Fe_2_O_3_, Fe_3_O_4_, Cr_2_O_3_, NiO, Cu_2_O, and Al_2_O_3_ forms on the inner surface of the kerf at the temperature of 1000–4000 K, isolating the metal from oxygen and hindering the cutting process. The slag is forced out by the forces of gravity, arc pressure, and surface tension. To remove the slag, it was proposed to reduce the surface tension of the slag through ionic dissolution and interaction of refractory oxides in Na_3_AlF_6_ cryolite when the change of Gibbs free energy occurs from −140 kJ to −300 kJ at the temperature of 4000 K.
4. Oxidizing cutting requires the composition of the gas mixture with high oxygen amount of up to 0.2–0.4 mole fraction at the temperature of 4000 K in the cutting zone, with electrons amounting up to 0.18–0.3 mole fraction at the temperature of 10,000 K, negative ions of oxygen and fluor less than 0.002 and 0.0018 mole fraction, enthalpy of more than 5 kJ/kg, heat capacity of more than 5.5 J/kg·K, and thermal conductivity of plasma more than 0.4 W/m·K at a temperature of 4000 K. These conditions are satisfied by the proposed oxidizing mixtures: 40–80% of KNO_3_, NaNO_3_, iron carbonate FeCO_3_, and 10–40% aluminum.
5. The exothermic effect allows regulating the thermophysical properties of plasma and can be used in underwater wet cutting. The introduction of 60% Fe_3_O_4_, MoO_2_, and WO_2_ and 20% Al, Mg, and Ti in exothermic termite mixtures improves plasma ionization, increases electrons amount of up to a 0.3-mole fraction at the temperature of 10,000 K, reduces the enthalpy of up to 1.5 kJ/kg and heat capacity of up to 4.5 J/kg·K, and increases the thermal conductivity of plasma of up to 1.16 W/m·K at the temperature of 4000 K.
6. Flux-cored wires with an oxidizing mixture are effective for cutting low-alloyed stainless steel as well as for cutting bronze and aluminum alloys. The optimum content of salts KNO_3_ and FeCO_3_ in the experimental wire was 50–60% with the introduction of 20–30% aluminum and 20% Na_3_AlF_6_. This composition allowed achieving stable cutting of 10KhSND steel with the kerf width of up to 4.7 mm, AISI 304L steel with the kerf width of up to 2.5 mm, and CuAl5 bronze with the kerf width of up to 3 mm with a deviation of ±0.3 mm.

## Figures and Tables

**Table 1:** Parameters of methods for underwater wet cutting of steels [6,7,8,9,10].

Method	Maximum Thickness of Steel, mm	Current, A	Voltage, V	Oxygen Consumption, m^3^/h	Cutting Speed for Steel, mm/min
Manual oxy-arc cutting using tubular electrodes of 8 mm	100	180–350	25–45	0.8–1	30–40 (for 10 mm)
Manual oxy-arc cutting using exothermic electrodes of 9.5 mm	35	150–300	25–45	12–60	400–500 (for 10 mm)
Manual arc cutting using coated electrodes of 4 mm	16	200–280	30–45	–	60–80 (for 10 mm)
Automatic plasma cutting	80	300–800	200–300	Air consumption of 0.06–0.12	300–400 (for 15 mm)
Automatic laser cutting	100	Power of 2–10 kW	–	Air consumption of 0.1–0.3 m^3^/h	300–1000 (for 15 mm)
Semi-automatic/automatic cutting using flux-cored wires of 2 mm	20	300–500	32–42	–	70–250 (for 10 mm)

**Table 2:** The composition of experimental flux-cored wires.

Wires	Flux-Core Composition, wt.%			
	KNO_3_	FeCO_3_	Na_3_AlF_6_	Al
PPR-APL1–1	30	30	20	20
PPR-APL1–2	25	25	20	30
PPR-APL1–3	35	35	20	10

**Table 3:** Parameters of underwater wet cutting.

Materials	Travel Speed, mm/min	Wire Feed Rate, m/min
10KhSND of 10 mm (3 plates)	50 (1–4 tests); 70 (5–7 tests); 100 (8–10 tests)	7; 8; 9; 10 (1–4 tests); 8; 9; 10 (5–7 tests); 8; 9; 10 (8–10 tests)
AISI 304L of 16 mm (1 plate)	70 (1–3 tests); 100 (4–6 tests)	8; 10; 12 (1–3 tests); 8; 10; 12 (4–6 tests)
CuAl5 of 10 mm (1 plate)	100 (1–3 tests)	10; 12; 14 (1–3 tests)
AlMg4.5Mn0.7 of 6 mm (1 plate)	200	5.5
AlMg4.5Mn0.7 of 12 mm (1 plate)	150	5.7

**Table 4:** Parameters of underwater wet cutting of 10 KhSND steel with PPR-APL1-1 wire.

Cut Number	I, A	U, V	Maximal Working Cycle Time, s	Maximal Idle Cycle Time, s	Short-Circuit Frequency, Hz
1	344 ± 80	38.7 ± 3.6	3.6	1.7	0.41
2	371 ± 67	38 ± 5.9	4.1	1.6	0.54
3	417 ± 43	36.5 ± 3.9	5	1.4	0.46
4	411 ± 35	36.7 ± 3	2	1.2	0.78
5	381 ± 43	38 ± 3.6	1.5	1.1	0.81
6	400 ± 50	36.7 ± 3	1.8	0.6	1.23
7	420 ± 26	36.3 ± 2.8	1.5	1.1	1.04
8	377 ± 35	38.1 ± 2.6	2.2	1.2	0.59
9	391 ± 34	37.5 ± 3.3	2.9	1.2	0.49
10	400 ± 31	37 ± 1.57	2.2	0.7	0.89

**Table 5:** Parameters of underwater wet cutting of AISI 304L steel with PPR-APL1-1 wire.

Cut Number	I, A	U, V	Maximal Working Cycle Time, s	Maximal Idle Cycle Time, s	Short-Circuit Frequency, Hz
1	350 ± 68	39.4 ± 5.4	3	1.2	1.3
2	377 ± 66	37.6 ± 6.3	0.6	0.3	2.4
3	389 ± 68	36.9 ± 6.1	0.5	0.28	3.1
4	368 ± 75	38.4 ± 5.9	2.5	0.8	0.8
5	391 ± 66	37.4 ± 5.5	1	0.53	1.8
6	402 ± 56	36.9 ± 3.1	0.5	0.5	2.2

**Table 6:** Parameters of underwater wet cutting of CuAl5 alloy with PPR-APL1-2 wire.

Cut Number	I, A	U, V	Maximal Working Cycle Time, s	Maximal Idle Cycle Time, s	Short-Circuit Frequency, Hz
1	360 ± 90	37.2 ± 4.5	2.3	0.24	1.53
2	375 ± 85	36.5 ± 3.5	0.4	0.28	2.27
3	406 ± 54	35.4 ± 1,4	0.6	0.35	2.93

**Table 7:** Hardness and HAZ width in underwater wet cutting of steels.

Steel	Travel Speed, mm/min	Wire Feed Rate, m/min	Hardness, HV		HAZ Width, mm
			Base Metal	HAZ	
10KhSND of 10 mm with PPR-APL1-1	100	10	132–157	116–164	4–5.2
10KhSND of 10 mm with PPR-APL1-2	100	10	130–163	107–177	4–4.5
10KhSND of 10 mm with PPR-APL1-3	100	10	132–158	106–168	6.2–7
AISI 304 of 16 mm with PPR-APL1-1	100	10	208–238	249–398	2–2.5

## Data Availability

Data sharing is not applicable.
